# Molecular and Therapeutic Aspects of Hyperbaric Oxygen Therapy in Neurological Conditions

**DOI:** 10.3390/biom10091247

**Published:** 2020-08-27

**Authors:** Inbar Fischer, Boaz Barak

**Affiliations:** 1 The Sagol School of Neuroscience, Tel Aviv University, Tel Aviv 69978, Israel; inbarfischer@mail.tau.ac.il; 2 The School of Psychological Sciences, Tel Aviv University, Tel Aviv 69978, Israel

**Keywords:** hyperbaric oxygen therapy, hypoxia, neurological conditions, neurodevelopmental disorders, autistic spectrum disorder, cerebral palsy, traumatic brain injury, mitochondria, white matter, reactive oxygen stress, angiogenesis

## Abstract

In hyperbaric oxygen therapy (HBOT), the subject is placed in a chamber containing 100% oxygen gas at a pressure of more than one atmosphere absolute. This treatment is used to hasten tissue recovery and improve its physiological aspects, by providing an increased supply of oxygen to the damaged tissue. In this review, we discuss the consequences of hypoxia, as well as the molecular and physiological processes that occur in subjects exposed to HBOT. We discuss the efficacy of HBOT in treating neurological conditions and neurodevelopmental disorders in both humans and animal models. We summarize by discussing the challenges in this field, and explore future directions that will allow the scientific community to better understand the molecular aspects and applications of HBOT for a wide variety of neurological conditions.

## 1. Introduction

### 1.1. Hypoxia in Neurological Conditions

The production of cellular energy by neurons and glial cells and the constant maintenance of brain metabolism depends on high consumption of oxygen [1]. Hypoxia is a condition in which an area receives an inadequate supply of oxygen, and is referred to as local hypoxia when it occurs in a specific tissue, such as brain tissue, or as generalized hypoxia when referring to the whole body [2]. It is known to cause a wide range of damage to the tissue, including cell death [3], induced inflammation processes [4], and impaired mitochondrial function [5].

Hypoxia in neurons may cause irreversible damage, interfere with their electrical communication [6], and cause significant damage to glial cells, especially oligodendrocytes (OLs) [1,7]. Hypoxia during developmental stages can lead to affected brain development and consequently, neurodevelopmental disorders (NDDs) [8,9]. Even after development, the brain consumes about 20% of the body’s overall oxygen consumption, which is only sufficient for its normal function, i.e., it consumes almost all of the energy that it receives [10]. Thus, when the brain suffers from any kind of damage or abnormality, the normal supply of oxygen may become insufficient, making oxygen a limiting factor in brain-damage recovery, in addition to other essential factors such as glucose levels, both key factors in neurological pathology.

Hypoxia is known to occur in a variety of neurological conditions, such as traumatic brain injury (TBI) [11,12], Alzheimer’s disease (AD) [13], stroke [14,15], cerebral palsy (CP) [16], multiple sclerosis [17] and autistic spectrum disorder (ASD) [6], and to be responsible for some of their symptoms. In a mouse model for AD, it was shown that hypoxia contributes to memory loss [18], and human subjects that experienced a hypoxic event, such as a stroke, were more prone to AD later in life [18]. Evidence of hypoxia in ASD subjects has been found, along with a reduction in cerebral blood flow (CBF) [6,19,20]. Although the role of hypoxia in ASD is still elusive, van Tilborg et al. [7] showed that upon postnatal hypoxia and fetal inflammation, rats exhibit social inhibition, increased repetitive and anxiety-like behaviors, and myelination deficits, which are all typical of ASD [21].

Options for treating hypoxia are limited. One study examined the use of long-term supplementation of lithium to treat rats affected by hypoxia and observed improvement in cerebral glucose metabolic rate in several brain regions after the treatment [22]. In another study, intranasal administration of C3a, a protein that is part of the complement system, reduced cognitive impairment in a mouse model of hypoxic–ischemic brain injury [23]. However, almost all known treatments involve a degree of invasiveness, and some are not yet fully understood in humans. Hyperbaric oxygen therapy (HBOT), a noninvasive treatment that is extensively used in humans for various conditions, has been found to improve therapeutic and molecular aspects in hypoxia-related conditions [24].

### 1.2. HBOT

In HBOT, the subject is placed in a chamber containing 100% oxygen gas at pressures of more than one atmosphere absolute (ATA); it is primarily used as a treatment for hypoxia-related conditions [24]. There are several treatment protocols, which vary according to the pathologies. For instance, treatment of diabetic wounds typically lasts for 30–40 days, at pressures of 2.0–2.4 ATA [25], whereas for stroke, in several studies the treatment that was used lasted for two months, with the subject being treated five days a week at a pressure of two ATA [26,27]. By providing a high-pressure oxygen-rich environment, HBOT increases the concentration of dissolved oxygen in the blood plasma and arterial oxygen pressure, which in turn might assist in supplying oxygen to hypoxic tissue [28,29].

The first hyperbaric chamber was constructed in 1662 by the British physician Nathaniel Henshaw. Unlike modern hyperbaric chambers, the first chambers used compressed air instead of pure oxygen [29] due to concerns regarding oxygen toxicity, which were later allayed. Traditionally, HBOT was used to hasten tissue recovery in wounds [30] and infections, aid in surgery such as cardiac surgery [31], alleviate carbon monoxide poisoning [32], as well as to treat other conditions. Since hypoxia was established as a key issue in several neurological conditions, HBOT was later studied in the context of neurobiological properties and was shown to ameliorate biological and behavioral deficits, as will be further discussed in this review. For example, in TBI, HBOT was found to improve CBF [33], restore impaired brain metabolism, as shown by increased cerebral adenosine triphosphate (ATP)-expression levels in a TBI rat model [34], decrease secondary cell death and neuroinflammation in a TBI rat model [35], and improve neuroplasticity [36]. In addition, improvement in memory and cognitive functions was found in treated subjects that suffered from chronic cognitive impairments [37] and in a treated AD mouse model [28]. Recently, HBOT has also been assessed for several NDDs, such as autism [6]. Although hypoxia’s role in these disorders is not well understood, HBOT was found to improve several of their pathological aspects.

Here, we will discuss the molecular effects of HBOT on different neurological conditions, as well as its therapeutic implications, focusing mainly on three neurological disorders: ASD, CP, and TBI.

## 2. HBOT from a Neurobiological Point of View

**Figure 1:**
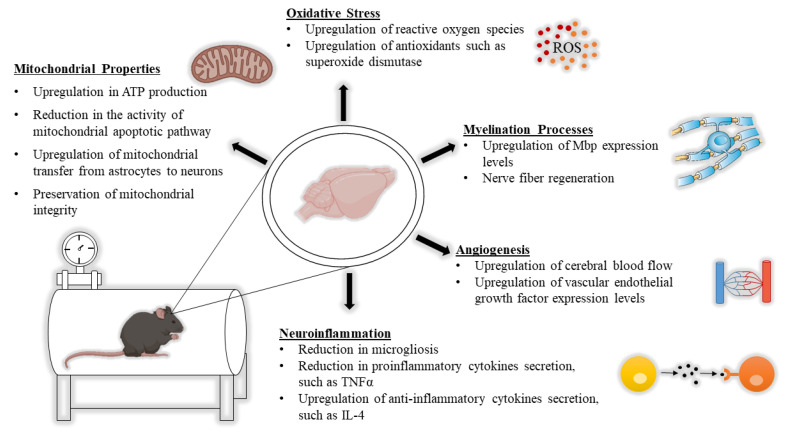
Key molecular changes in the brain following hyperbaric oxygen therapy (HBOT). Mitochondrial properties, oxidative stress, myelination processes, angiogenesis, and neuroinflammation are all altered following HBOT.

HBOT has been observed to cause molecular changes in the central nervous system (CNS) of subjects afflicted with neurological conditions, including changes in mitochondrial function, white matter, neuroinflammation, oxidative stress, and CBF. In this section, we discuss HBOT’s mechanism from a neurobiological point of view and present some recent studies in this field (Figure 1).

### 2.1. The Impact of HBOT on Mitochondrial Properties

The mitochondrion is a vital cell organelle that is responsible for supplying energy to cells by generating ATP [38]. It also mediates cellular processes such as apoptosis [39] and proliferation [40], and takes part in neuronal functions such as synaptic plasticity [41].

The mitochondria produce ATP via an electron-transport chain, in which oxygen has an important role as the last electron acceptor in the chain. In this process, a proton gradient is created as protons are pumped from the mitochondrial matrix to the intermembrane space. The high concentration of protons in the intermembrane space creates an electrical potential and a chemical gradient of protons in the membrane, which is vital for maintaining membrane potential and for the ATP-production process. When the membrane’s integrity (i.e., its structure or function) is compromised, an apoptotic pathway can be initiated inside the cell.

In neurons, there is a great need for well-functioning mitochondria, as neuronal activity consumes a large amount of energy and neurons have only small energy reserves [42,43]. While there are several conditions that may cause mitochondrial dysfunction, such as mutations in the mitochondrial DNA that amplify with age [44], this organelle’s function relies heavily on oxygen consumption. Reduction in oxygen levels, such as a hypoxic state, can damage energy production [45] and cause lactate aggregation in the tissue as well as other metabolic changes [8]. Accordingly, several studies have considered the use of HBOT to treat neuronal conditions related to mitochondrial dysfunction, by increasing the amount of oxygen arriving to the mitochondria.

**Figure 2:**
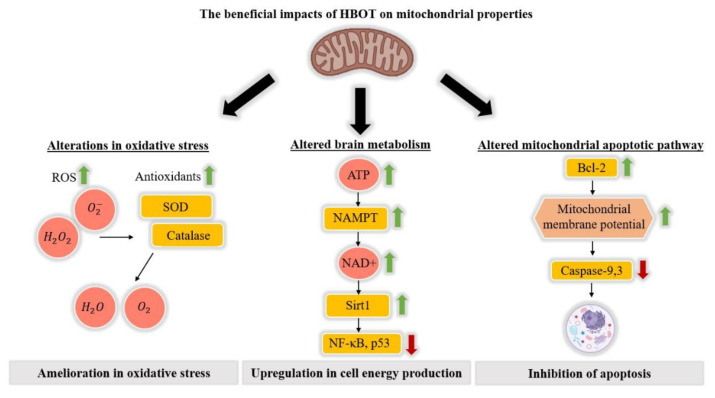
The beneficial impacts of HBOT on mitochondrial properties. Three key processes related to mitochondria that are affected by HBOT- oxidative stress, cell energy production, and apoptosis.

HBOT was found to facilitate the correction of mitochondrial abnormalities such as those in mitochondrial metabolism [46], improve the integrity of compromised mitochondrial membranes [47], and inhibit secondary cell death by causing the transfer of mitochondria from astrocytes to neurons [48]. Some studies measured HBOT’s impact according to changes in ATP levels after treatment [34,46,49]. For example, Hu et al. [46] studied a rat model for strokes, induced by middle cerebral artery occlusion combined with hyperglycemia to induce ischemia and hemorrhagic transformation. Following HBOT, ATP-expression levels were measured by the enzyme-linked immunosorbent assay (ELISA) and the results showed significantly upregulated ATP expression, along with an increase in NAD+ expression, an important marker of energy metabolism. In addition, they observed an increase in nicotinamide phosphoribosyltransferase (NAMPT) activity, which is a production-limiting protein of NAD+, and upregulation of Sirt1 expression, which is an upstream protein of p53 and NF-κB, the latter related to cell apoptosis and inflammation, respectively (Figure 2).

Concomitant reduction in the expression of p53 and NF-κB strengthened the fact that an ATP/NAD+/Sirt1 pathway had been activated by HBOT. Furthermore, administration of NAD+ exhibited an effect similar to that of HBOT, and administration of ATP synthase inhibitor, NAMPT inhibitor, or Sirt1 small interfering RNA (siRNA) prevented HBOT‘s positive effect. Activation of this specific pathway by HBOT reduced cell necrosis and improved neurological function [46].

Additional studies considered other aspects of HBOT’s effects on mitochondria. Palzur et al. [47] examined HBOT’s effects on mitochondrial integrity and the activity of the mitochondrial apoptotic pathway in a rat model of TBI. They created lesions on a single hemisphere and used the uninjured hemisphere for comparison. A statistical analysis was performed comparing three different groups: A control group that was not treated with HBOT and went through dynamic cortical deformation in one hemisphere, a HBOT-treated group that went through the same procedure as the first group, and a sham group that underwent surgery without dynamic cortical deformation. The results showed that mitochondrial integrity, measured by mitochondrial transmembrane potential, was significantly restored after HBOT, as seen by lower differences in transmembrane potential between both hemispheres in the treated group compared to the untreated control group. Furthermore, in the untreated control group, activity of both caspase-3 and caspase-9, proteins known to mediate apoptosis through the mitochondria’s apoptotic pathway, was increased as compared to the treated group in which their activity was significantly reduced. However, caspase-8, a protein known to initiate apoptosis, showed no significant difference in activity between the treated and untreated groups. These results were consistent with the previous work, in which an increase in the expression of proteins Bcl-2 and Bcl-xL, both of which inhibit apoptosis, was measured after HBOT in the penumbra of a TBI rat model [50]. In addition, no significant change was measured in the amount of the proapoptotic protein Bax [50]. Together, these studies strongly indicate that HBOT specifically influences the mitochondrion’s intrinsic apoptotic pathway, as Bcl-2, caspase-3, and caspase-9 are involved in this particular pathway, whereas caspase-8 is mostly involved in the extrinsic apoptotic pathway [51,52] (Figure 2).

Lastly, an in vitro study was performed to determine HBOT’s effect on mitochondrial transfer from astrocytes to neurons [48]. The researchers placed primary rat neuronal cells, co-cultured with astrocytes, in a HBOT chamber, and then exposed the cells to either tumor necrosis factor-alpha (TNF-α) or lipopolysaccharide to create a brain injury or stroke-like environment during the secondary cell death stage. They observed that the preliminary use of HBOT increases cell viability and induces higher mitochondrial transfer from astrocytes to neurons, suggesting that mitochondrial transfer, activated by HBOT, protects neurons from secondary cell death [48].

Today, there are many studies focusing on the effects of HBOT on mitochondrial properties in the context of TBI and stroke. Mitochondrial impairment is also a common phenomenon in NDDs such as ASD [53,54], and thus a natural direction for future research would be to examine HBOT’s effectiveness on those disorders as well.

### 2.2. The Impact of HBOT on Alterations in White Matter

Myelin is the outer layer that envelopes neurons [55]. It is comprised mostly of lipids and is produced by OLs in the CNS [56]. Myelin plays an important role in isolating the electrical information passing through an axon, which is crucial for neuronal connections [55,57]. This isolation layer is key to the proper transmission of information between neurons, as evidenced by neurological conditions that include myelination deficits [58,59,60,61,62,63].

Evidence from numerous studies suggests a connection between hypoxia and myelination deficits [7,64,65]. Back et al. discovered that late OL precursors are extra-susceptible to oxygen deficiency, as reflected by high cell death [65]. Hence, hypoxia may result in a reduction in the number of cells differentiated to mature myelinating OLs. In Cree et al. [60], administration of the drug clemastine to hypoxic injured mice resulted in an elevated number of myelinating OLs and improved myelin ultrastructure. Together, these findings suggest that HBOT can ameliorate the damage caused by hypoxia to white matter, and might ease some of the symptoms in neurological disorders related to white matter deficiency and hypoxia.

One work investigated the effect of HBOT on nerve fiber regeneration in human TBI subjects with post-concussion syndrome [66], which is a set of symptoms that are common after TBI. The effects were observed using diffusion tensor imaging (DTI) [67], a magnetic resonance imaging (MRI) technique that can assess changes in white matter in live human subjects. Along with major improvements in clinical symptoms, they observed an increase in the fractional anisotropy, a value that represents the degree of anisotropy in the diffusion of a voxel, in diverse brain regions such as the corpus callosum, internal capsule, and midbrain. They also observed decreased mean diffusivity, a value that represents the total diffusion in a voxel, mainly in the white matter of the frontal lobe [66], both indicative of restored neuronal fibers.

In animal models, several studies have investigated HBOT’s influence on myelin basic protein (Mbp) and remyelination. Baratz-Goldstein et al. [68] studied these effects in a mouse model of mild TBI, applying either immediate or delayed HBOT treatment. They found that both treatments ameliorated the reduced Mbp expression and demyelination compared to the untreated control group, and that both the treated groups and the sham group had the same measured amount of Mbp expression [68]. Differences between the treated groups and the control group were already observed 10 days after the beginning of the treatment, meaning that HBOT led to a rather fast recovery, even when limiting the length of the treatment to four days. In Kraitsy et al.’s work [69], HBOT resulted in upregulation of the expression levels of two specific isoforms of Mbp which are important to myelin structure, upregulation of proteolipid protein (Plp), and increased remyelination processes.

Although these and other studies show the important effects of HBOT on white matter in the nervous system, there is a lack of mechanistic research performed directly on OLs, which could be key to understanding the mechanism acting in HBOT and myelination processes.

### 2.3. The Impact of HBOT on Neuroinflammation

Neuroinflammation in the brain is a reaction of the CNS immune cells and the peripheral immune system when the brain experiences trauma, injury or other pathological conditions. It is mainly created by microglia and astrocytes [70,71], or by infiltrating peripheral immune system cells when the blood–brain barrier is compromised [72], and those cells secrete various proinflammatory cytokines [71,73]. While neuroinflammation is a necessary process for tissue repair, a prolonged state of hyperactivity of the immune system, usually referred to as chronic inflammation, may cause extensive tissue damage, as can happen in AD [74], autism [75,76,77], CP [78,79], and TBI [80].

Hypoxia is a major factor in inducing neuroinflammation, whereby it mediates the activation of cells from the innate immune system, resulting in upregulated secretion of proinflammatory cytokines and increased immune cell aggregation [4,81,82]. On the other hand, chronic neuroinflammation can also cause or increase a hypoxic state, as evidenced by the upregulated expression of hypoxia-inducible factor 1-alpha (Hif1-α), thereby creating a positive-feedback cycle between the two conditions [83,84].

One of the neurodegenerative disorders that is highly associated with neuroinflammation is AD. In AD, large amounts of amyloid plaques accumulate in the brain. These plaques may incur microgliosis, in which activated microglia surround the plaques and secrete proinflammatory cytokines [85,86,87]. Shapira et al. [28], studied the influence of HBOT on an AD mouse model and found less microglia around the amyloid plaques after the treatment, along with less proinflammatory cytokines such as TNF-α, as shown by immunofluorescence staining in the hippocampus. Those researchers suggested that the reduction in the amount of cytokines might be due to the reduced amount of microglia. Moreover, a morphological examination showed that HBOT enhances microglial process extension and increases the number of sprouting microglia around the plaques, which could indicate a change in the microglia’s activation state and function. These findings emerged in parallel to a reduction in hypoxia levels and in the amount of amyloid plaques, and improvement in cognitive- and anxiety-like-related behaviors [28].

Several studies have found that aside from the decrement in the secretion of proinflammatory cytokines, HBOT attenuates neuroinflammation via enhancement of immune cell secretion of anti-inflammatory cytokines [28,88,89]. In animal models, HBOT increased the expression levels of mRNA encoding the anti-inflammatory cytokine interleukin (IL-4), as measured by real-time quantitative PCR [28], and increased the concentration of the anti-inflammatory cytokine IL-10 in the cortices, as measured by ELISA [88]. Changes in anti-inflammatory cytokines were found in human patients with various neurological conditions as well [89].

The anti-inflammatory effect of HBOT can promote tissue repair and prevent secondary cell death by hindering the apoptotic pathway. This was shown in a TBI mouse model, in which increased expression of the anti-inflammatory cytokine IL-10 following HBOT resulted in reduced caspase-3 activity, along with a reduction in Bax-expression levels [88]. Together, these findings suggest that HBOT has the ability to not only stop future damage, but also to initiate a healing process in the tissue.

### 2.4. The Impact of HBOT on Oxidative Stress

Oxidative stress is the result of an imbalance of reactive oxygen species (ROS) inside the cell. Oxidative stress can have several causes, a primary one being the release of ROS from the mitochondria in a hyperoxic state [90,91]. Although ROS can be beneficial under normal conditions to several cell processes, such as regulation of synaptic plasticity [92] and molecular signaling [93], abnormal ROS levels can cause DNA fragmentation and crosslinking of proteins, which can lead to cell apoptosis [93,94]. One of the greatest concerns in using HBOT is an increase in oxidative stress, because of the high administration of oxygen and the potential for hyperoxia.

There are conflicting results on HBOT’s effect on oxidative stress; some studies have shown that after HBOT, there is an increase in antioxidants which are highly important for balancing ROS concentration [95,96]; others have indicated an increase in ROS and induction of oxidative stress, in accordance with the higher oxygen supply [97,98,99]. However, some of the latter studies were performed with either repeated consecutive exposures [99], which deviates from the standard protocol, or with the relatively high baric pressure of four bars [97], performed on animals, which is higher than the clinically approved pressure levels. In addition, it was shown that 24 h after the end of the treatment, the elevated ROS returned to baseline [100,101], and the DNA damage incurred by the elevation in ROS was reversible [102].

Despite the rise in ROS, several studies on cerebral ischemia and other conditions have shown that after HBOT, there is an increase in a variety of antioxidants [95,96]. Both Wada et al. [96] and Nie et al. [103] demonstrated an increase in antioxidants such as superoxide dismutase (SOD) and catalase—important antioxidant enzymes—mediated by HBOT preconditioning, in rodent models for cerebral ischemia and spinal ischemia, respectively.

As already noted, an increase in ROS can result in positive outcomes, and not only cell toxicity. ROS may assist in initiating signaling in cellular pathways involved in angiogenesis via stabilization of Hif1-α, which in turn increases the production of vascular endothelial growth factor (VEGF) [104,105,106]. This further solidifies the relationship between HBOT and angiogenesis, which is discussed in detail in Section 2.5.

Although HBOT can increase ROS concentrations, its use with the right protocol has been established as safe [101,107]. Furthermore, the aforementioned elevation in antioxidants may counteract this increase in ROS, creating a balance in which the oxidative stress state remains at the same level as prior to treatment. Wada et al. [96] suggested that ROS upregulation may enhance the expression of antioxidants such as SOD, and even create ischemic tolerance (Figure 2).

### 2.5. The Impact of HBOT on Induction of Angiogenesis and Changes in CBF

When the blood supply to a certain brain area is reduced, the high oxygen demand in that region might not be met, which could lead to a hypoxic state. In autism, for example, reduction in CBF was found in specific brain regions related to language, such as the temporal lobes [6], and to occur during specific activities, such as observing facial expressions [108].

The therapeutic value of HBOT in increasing CBF and angiogenesis—the process by which new blood vessels are created—in wounds has been firmly established [105]. HBOT’s effect on these aspects in neurological conditions has been studied mainly by using various imaging techniques, such as positron emission tomography (PET) [109], single-photon emission computed tomography (SPECT), and functional MRI [110]. Most of the studies performed on humans used SPECT imaging and found increased blood flow, along with symptom improvement, in various neurological conditions [27,33,36].

Tal et al. [66] observed increased CBF and cerebral blood volume after HBOT in human TBI subjects, as measured by dynamic susceptibility contrast enhancement MRI imaging, potentially indicating angiogenesis. In other works, evidence of induction of angiogenesis by HBOT was found through the investigation of molecular markers. Two studies that measured such markers in the CNS [111,112] showed upregulation of VEGF mRNA, which is responsible for, among other things, vascularization. Angiogenesis could be a central mechanism by which HBOT increases CBF, as well as vascular repair.

Overall, the discussed studies have shown that HBOT can assist in vascular repair. In addition to achieve a direct supply of oxygen, there are strong indications that HBOT can indirectly increase the amount of oxygen reaching a tissue by encouraging angiogenesis in the CNS.

## 3. HBOT from a Therapeutic Point of View

As was discussed in Section 2, HBOT has broad beneficial molecular effects in neurological conditions, emphasizing this treatment’s exciting therapeutic potential. In the following, we review HBOT from a therapeutic perspective in human clinical studies focused on neurological conditions.

### 3.1. ASD

ASD is a group of developmental disorders that are characterized by inhibited social behavior and anxiety, among other symptoms [21]. According to the Centers for Disease Control and Prevention, one out of 54 children are diagnosed with ASD [113], making it one of the most common NDDs. No efficient treatment has been found, and the etiology of this disorder is mostly unknown. Interestingly, several of the molecular abnormalities exhibited by ASD, such as mitochondrial dysfunction [53,54], white matter alterations [114,115], and hypoperfusion [116], are associated with dysfunctions that are potentially treatable by HBOT. Therefore, there is hope that HBOT will potentially ameliorate some symptoms of ASD [84,117].

Over the years, studies investigating HBOT’s effects on ASD symptoms have provided conflicting results [118,119,120,121,122,123]. In one study [118], 62 children with ASD were either treated with HBOT (oxygen level 24%, pressure level 1.5 ATA) or were assigned to an untreated control sham group. The treatment group showed major improvements in language skills and social interaction, as measured by a Clinical Global Impression assessment performed by a physician, along with other improvements.

A different study by Rossignol et al. examined the efficacy of two different HBOT protocols: 1.5 ATA with 100% oxygen or 1.3 ATA with 24% oxygen [123]. Behavioral improvements were assessed by the subjects’ parents according to validated questionnaires (ABC-C, SRS, ATEC). In both groups, there was a decrease in inflammation, measured by decreased C-reactive protein expression levels, and improvement in clinical aspects including speech and cognitive awareness, compared to before the treatment. We note that this study was an open-label trial, in which the subjects were not randomly assigned to their groups. Three more studies found clinical and behavioral improvements after HBOT that included social and cognitive improvements [124,125,126]. Unfortunately, those studies had no control group, complicating the interpretation of their results.

Moreover, some studies investigating HBOT in the context of ASD detected no major improvement in the aspects mentioned above [119,120,127]. In a study performed by Granpeesheh et al., human ASD subjects were divided into two groups: One was subjected to 24% oxygen at 1.3 ATA, and the other, a sham group, was subjected to one ATA of room air (21% oxygen) [119]. No significant difference in behavioral aspects was found according to validated psychological tests (e.g., ABC, ADOS, SRS). A controlled study by Sampanthavivat et al. revealed a significant improvement in ASD children’s behavior in both the treated group (1.5 ATA, 100% oxygen) and a sham group with the slightly elevated pressure of 1.15 ATA in room air [127]. No significant difference was found between the two groups in most aspects, although in several behavioral tests, the evaluations by clinicians and parents did not match. We note that in several studies of HBOT, the use of slightly elevated pressure has been observed to constitute some form of treatment [128]; therefore, its use in the treatment of a control group could constitute a problem. We discuss this problem further in Section 3.2 and Section 4.

A work by Luo et al. studied HBOT’s effect on a mouse model of Fragile X syndrome, which is a disorder that is strongly related to ASD, and found improvements in social and anxiety-like behavior [122].

As ASD is quite a large spectrum, we believe that more fine-tuned research is required in this field, focusing on specific groups on the spectrum with potentially similar etiology. In particular, ASD subpopulations that are known to have mitochondrial dysfunction constitute 5% of the overall ASD population and have certain shared clinical and molecular characteristics [53,129]. This makes them prime candidates for such studies, in no small part because HBOT has been shown to have a positive influence on mitochondrial activity, as discussed in Section 2.1.

### 3.2. CP

CP is a NDD that occurs mainly due to brain injury during cerebral development. It is considered to be a nonprogressive movement disorder that is characterized by motor deficiency such as tremor and muscle weakness. Most of the cases of CP occur during the prenatal phase, although it may occur any time before the brain is fully developed [16].

The fact that the main cause of CP is reduced CBF [130,131] indicates its strong connection to hypoxia [16], and therefore the therapeutic potential of HBOT. Indeed, HBOT has been tested as a noninvasive therapy for subjects with CP. Unfortunately, the vast majority of the research done in this field has shown data that do not support a therapeutic effect of HBOT on CP [132]. In Collet et al., [133] 111 children with CP participated in a study in which the treated group was placed in an environment with 1.75 ATA pressure and 100% oxygen, versus the control group, which was subjected to a pressure of 1.3 ATA and room air. No significant differences were found between the control and treated groups with respect to motor or cognitive properties; however, both groups showed improvement in those properties over time, as compared to the situation before exposure to the high pressure. Therefore, it could be argued that the control group improved because of the effect of the slightly elevated pressure, meaning that HBOT could have a positive therapeutic effect on CP. Indeed, Boussi-Gross et al. [36] discussed the effects of placing human subjects in 1.3 ATA and room air and the problems that arise in applying these conditions to control groups, as they cause observable physiological changes, such as a 50% increase in tissue oxygen levels. They suggested alternative ways of designing control group protocols which have a placebo effect while avoiding “accidental” treatment.

A more recent study investigated the combination of HBOT and intensive rehabilitation that included physical therapy and speech therapy, among others, compared to a control group that was treated only with intensive rehabilitation [128]. The study included three hyperbaric treatments which differed in pressure and oxygen levels. All three treatments resulted in significantly improved motor function compared to controls, indicating that hyperbaric treatment could be beneficial for CP. We note that one of the improved groups was treated with 1.3 ATA and room air, which is consistent with the improvement observed in the control group of Collet et al. [133].

We end this section with two observations which, in our opinion, warrant further investigation. First, we note that most studies were done on a relatively wide range of ages. CP is considered to be a nonprogressive condition, and therefore it might be that, as in ASD, there is a critical age for treating it. Second, the beneficial effects of HBOT’s 100% oxygen level versus treatment only with elevated pressure levels and room air should be further studied, following the work of Mukherjee, in which a group that was treated only with elevated pressure showed the same improvement as two other groups that were subjected to both high pressure and 100% oxygen [128].

### 3.3. TBI

TBI is one of the leading causes of death and disability among young people [11]. Complications from TBI can occur even years after the injury, such as post-concussion syndrome, in which severe headaches and other symptoms appear [11]. Hypoxia and TBI are strongly tied when cerebral blood vessels are damaged, hence HBOT has been extensively studied as a potential treatment for TBI [134,135,136].

HBOT was shown to have a major therapeutic value for TBI in several preclinical studies [68,137] and clinical studies [36,66,138,139,140,141,142]. In Boussi-Gross et al. [36] HBOT improved cognitive function, as well as the quality of life, of human TBI subjects, in a randomized controlled trial consisting of 56 TBI subjects. This study utilized a crossover-based control group, in which there were two experimental groups: The first was treated during the first two months of the experiment, and the second was treated in the following two months. Each group was compared to itself before and after treatment, and the groups were compared after the first group completed its treatment and before that of the latter group has begun. Three different parameters were measured before and after treatment: Cognitive function—which was measured with four different cognitive indices, quality of life—measured with a questionnaire, and brain activity—measured by SPECT. All measurements showed a significant improvement in memory, attention, and cerebral perfusion. Those improvements were found only in the treated group and the crossover group after HBOT, and not during the control untreated period. The subjects underwent HBOT years after their injuries, potentially indicating that in the case of TBI, there is no critical period for treatment.

In Rockswold et al. [142], 42 human subjects with TBI were randomly divided into two groups: Three consecutive days of HBOT combined with normobaric oxygen therapy (NBOT), or an untreated control group. Along with a significant reduction in lactate-to-pyruvate ratio, intracranial pressure and other physiological aspects, a reduction in mortality rate in the HBOT/NBOT group was found, and a significant improvement in the Glasgow Outcome Scale was also seen at a six-month follow-up. These results are consistent with the findings of Rockswold et al. in which 168 human subjects with TBI were randomly divided into a two-week HBOT group or an untreated control group, and a significant reduction in mortality rate was observed in the treated group [139]. Moreover, in both Tal et al. [66] and Tal et al. [140], a retrospective analysis of HBOT for human TBI subjects revealed enhanced CBF along with cognitive improvements such as in motor skills and processing speed [66,140].

In the 40 years of extensive research on HBOT’s effects on TBI, focusing on both molecular and therapeutic aspects, it seems that many studies support the use of HBOT for TBI [135,136]. However, most clinical studies of human TBI subjects treated with HBOT did not have an appropriate sham group, and the treatment efficacy for human subjects should be further investigated [141]. The issue of constructing a proper sham group in TBI studies could be challenging due to the fact that in many cases immediate care is necessary and due to the inherent heterogeneity of TBI cases. It is interesting to note that CP is usually caused by a brain injury during developmental stages, but unlike TBI, more conflicting results were found regarding HBOT’s treatment efficacy. This calls for a consideration of the differences in protocols between treatments of the two conditions, and whether any insight can be gained from a successful HBOT for TBI, despite the many differences between the two conditions.

## 4. Discussion and Future Research

In conclusion, there is an abundance of evidence for HBOT’s improvement of neurological conditions in both molecular and therapeutic aspects. The main molecular changes that have been observed consist of improvements in myelination processes and mitochondrial activity, enhancement of angiogenesis, and a decrease in neuroinflammation. Therapeutic aspects include improvement of memory, cognitive and motor functions, and quality of life.

On the other hand, questions remain that require further investigation. One of the exciting scientific questions is how the myelination process and mitochondrial activity, both known to be independently affected by HBOT, are linked. The relationship between OLs and mitochondrial activity has been examined, and it was shown that mitochondrial abnormalities can influence OL differentiation, maturation, and vitality [143,144]. Hence, future research could explore the deeper mechanism governing the interaction between those molecular changes in the context of HBOT, and particularly whether improvement in white matter integrity occurs due to improvement of mitochondrial activity. In addition, the connection between the molecular mechanisms and therapeutic aspects should be examined, for example, by finding an association between brain areas that experience molecular changes and the clinical effects on the subject.

Apart from a few studies in which HBOT caused seizures [107] and other side effects [145], HBOT has been found to be safe for use in neurological conditions. It is important to note that in certain protocols, especially ones with very high pressure (more than three ATA), HBOT has the potential to increase oxidative stress to harmful levels. Therefore, only certain HBOT protocols should be used for neurological conditions, with a consideration of the most effective and safe choice for each condition and pathology.

The considerable variation in HBOT protocols and in the design of control groups makes it hard to compare the conflicting results in this field. Moreover, many of these studies also vary greatly in the age of their subjects. Since NDDs influence patients at critical stages of their development, the critical times for treatment should be examined in future research.

A key issue in HBOT research is how to design a suitable control group that achieves a “placebo effect” but does not confer real treatment or real physiological changes. To overcome this issue, several types of sham groups have been considered. Some studies used an HBOT chamber with 1.3 ATA and room air for the sham group. As already noted, even though it may seem that this small elevation in pressure causes no physiological changes, it can cause a 50% increase in oxygen in the tissue and therefore, it does constitute some form of effective treatment. Other solutions that have been considered are simulating the feeling of high pressure by frequent alternations between high and low pressure, or supplementing compressed air with lower oxygen concentrations. However, those approaches encounter both technical difficulties and ethical issues. Mukherjee’s study, discussed in Section 3.2, suggested a method that bypasses those difficulties [128]. In that study, the control group went through a rehabilitation treatment while the experimental group went through both HBOT and rehabilitation. In that manner, they isolated the impact of HBOT on the efficacy of the treatment. We feel that examining different types of control groups and then choosing one to become the “conventional” control group would significantly enhance our ability to understand the functions of HBOT.
